# NK3R Mediates the EGF-Induced SLα Secretion and mRNA Expression in Grass Carp Pituitary

**DOI:** 10.3390/ijms20010091

**Published:** 2018-12-26

**Authors:** Xiangfeng Qin, Cheng Ye, Xiaoyun Zhou, Jingyi Jia, Shaohua Xu, Qiongyao Hu, Guangfu Hu

**Affiliations:** College of Fisheries, Hubei Province Engineering Laboratory for Pond Aquaculture, Huazhong Agricultural University, Wuhan 430070, China; qinxiangfeng@webmail.hzau.edu.cn (X.Q.); m15355772353@163.com (C.Y.); zhouxy@mail.hzau.edu.cn (X.Z.); jiajy@163.com (J.J.); xsh9593@163.com (S.X.); HQY960819@163.com (Q.H.)

**Keywords:** NK3R, EGF, SLα, NKB, pituitary, grass carp

## Abstract

Epidermal growth factor (EGF) is a potent regulator of cell function in many cell types. In mammals, the EGF/EGFR system played an important role in both pituitary physiology and pathology. However, it is not clear about the pituitary action of EGF in lower vertebrates. In this study, using grass carp as a model, we found that EGF could stimulate NK3R mRNA and protein expression through pituitary ErbB1 and ErbB2 coupled to MEK/ERK and PI3K/Akt/mTOR pathways. In addition, EGF could also induce pituitary somatolactin α (SLα) secretion and mRNA expression in a dose- and time-dependent manner in vivo and in vitro. The stimulatory actions of EGF on SLα mRNA expression were also mediated by PI3K/Akt/mTOR and MEK/ERK pathways coupled to ErbB1 and ErbB2 activation. Our previous study has reported that neurokinin B (NKB) could also induce SLα secretion and mRNA expression in carp pituitary cells. In the present study, interestingly, we found that EGF could significantly enhance NKB-induced SLα mRNA expression. Further studies found that NK3R antagonist SB222200 could block EGF-induced SLα mRNA expression, indicating an NK3R requirement. Furthermore, cAMP/PKA inhibitors and PLC/PKC inhibitors could both abolish EGF- and EGF+NKB-induced SLα mRNA expression, which further supported that EGF-induced SLα mRNA expression is NK3R dependent.

## 1. Introduction

Epidermal growth factor (EGF) is a member of EGF family that also includes other EGF-like ligands, such as heparin-binding EGF-like growth factor (HB-EGF), transforming growth factor α (TGF α) and betacellulin (BTC). EGF, acting through EGF-receptors (ErbB), is a potent modulator of cell proliferation/differentiation in a wide variety of tissues. In mammals, expression of EGF and ErbBs have been detected in both the normal pituitary and pituitary adenomas, and the EGF/ErbB system has been explored in both pituitary hormone regulation and pituitary cell proliferation/differentiation [1,2,3,4,5,6,7,8]. Previous studies have reported that ErbBs and its ligands are all abundantly expressed in pituitary corticotroph cells [9], and functional studies confirm a role of EGF/ErbB system in corticotroph proliferation and hormone secretion both in a direct and paracrine fashion [10]. In addition, most EGF-secreting cells were also identified in luteinizing hormone (LH)-positive gonadotrophs [11]. In primary cultured pituitary cells, EGF enhances LH release [12] while in turn gonadotropin hormone releasing hormone (GnRH) stimulation further increases EGF secretion, in a positive feedback mechanism. Finally, ErbB and its ligands are also detected in lacto-somatotroph cells and have a functional role in prolactin (PRL) regulation at both gene and protein levels [13,14,15], thus far, EGF has been shown to have little effect on growth hormone (GH) regulation [16]. In zebrafish, EGF and ErbB are both distributed in the pituitary, but they do not have effects on follicle stimulating hormone β (FSHβ), LHβ and GH mRNA expression in primary cultured pituitary cells [17]. But beyond that, little is known about the functional role of EGF in other pituitary hormone secretion and mRNA expression in teleost.

Somatolactin (SL), the last member of the GH/PRL family, is a fish-specific hormone released from the neurointermediate lobe (NIL) of the posterior pituitary [18]. In the previous studies, two isoforms of SL (SLα and SLβ) have been identified in fish pituitary [19,20,21]. To date, SL has been shown to be involved in diverse functions in fish models, including reproduction [20], stress response [22], lipid metabolism [23] and osmoregulation [24]. In our recent studies, the TAC3 gene products, neurokinin B (NKB) and NKB-related peptide (NKBRP), were both found to stimulate SLα secretion and mRNA expression in grass carp pituitary cells via NK3 receptor (NK3R) activation [25]. Although NK3R is known to be important in reproductive functions in mammals [26,27,28] and teleost [29,30,31], no information is available for NK3R regulation in the fish model, especially at the pituitary level as a regulatory target for modulation of pituitary hormone secretion/gene expression.

In mammals, function crosstalk between ErbBs and G-protein coupled receptors (GPCR) have been reviewed in cancer tissues and cell lines [32]. The co-overexpression/activation of ErbBs and GPCRs in several tumor types has led to a consensus of a cross talk among these receptors. Now, two models have been proposed: (1) GPCR uses the ErbB to mediate its biological effect, and (2) EGF uses GPCR to mediate its effects. Although bidirectional interactions between GPCR and EGF signaling have been characterized in several cancer cells, the status of their interactions in the pituitary is unknown. In the present study, using grass carp pituitary cells as a model, we found that in the presence of EGF, and NKB-induced SLα mRNA expression is enhanced. This observation leads us to hypothesize that EGF and NKB may share a common pathway in the control of SLα gene expression, and that EGF may use NK3R to mediate its biological effects. Our objectives were to: (i) Characterize EGF-induced SLα release and gene expression, (ii) determine whether this effect requires NK3R.

## 2. Results

### 2.1. Molecular Cloning and Tissue Distribution of Grass Carp EGF and EGFR

**Figure 1:**
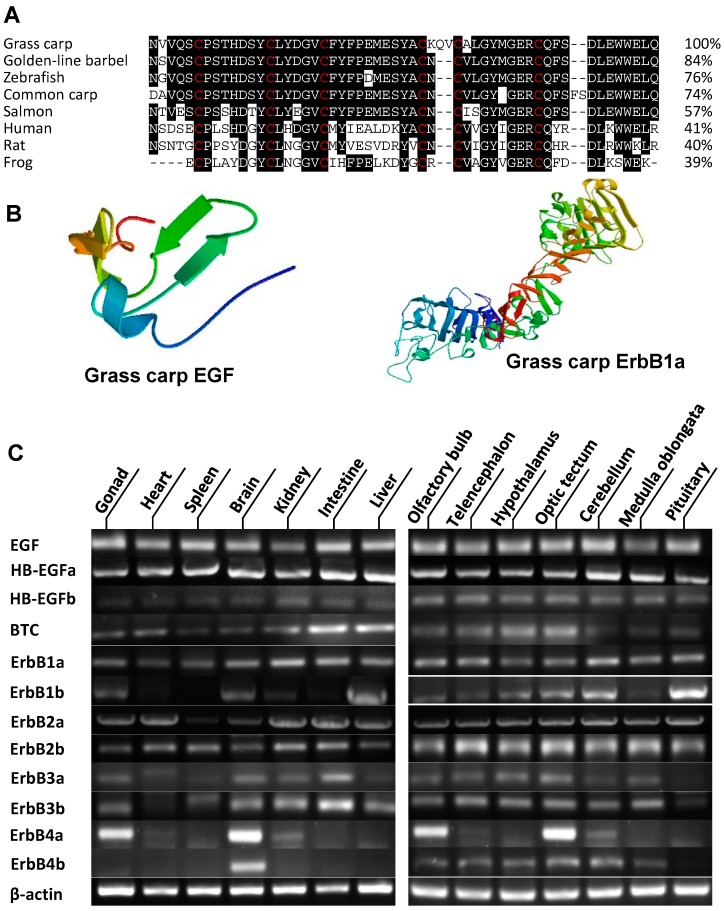
**Sequence analysis and tissue distribution of grass carp epidermal growth factor** (**EGF) and its receptors ErbBs.** (**A**) Amino acid sequence alignment of grass carp EGF mature peptide with that of other vertebrates using the Clustal-W algorithm with Mac Vector program. The conserved amino acid residues in these sequences are shadowed in black. The disulfide bond across cysteine residues is indicated in red color. (**B**) The three-dimensional protein models of grass carp EGF and ErbB1a. (**C**) Transcript expression of EGF, heparin-binding EGF-like growth factor (HB-EGFs), betacellulin (BTC) and ErbBs in various brain regions of grass carp. Total RNA was isolated from various tissues and brain areas in grass carp and subjected to RT-PCR using primers specific for grass carp EGF, BTC, HB-EGFa, HB-EGFb, ErbB1a, ErbB1b, ErbB2a, ErbB2b, ErbB3a, ErbB3b, ErbB4a, and ErbB4b, respectively. Parallel RT-PCR for β-actin was also conducted to serve as the internal control.

The full-length EGF (GenBank No.: MH161172) was isolated from grass carp pituitary. Sequence analysis showed that grass carp EGF cDNA is 3654 bp in length encoding 1218 amino acid (Supplemental Figure S1). Similar to other vertebrates, the mature EGF peptide (55 amino acids) in grass carp is located near the transmembrane domain, and it shares homology with mature EGF peptide from zebrafish (76%), human (41%) and rats (40%) (Figure 1A). In addition, grass carp mature EGF peptide also contains six cysteine residues, which is critical for the formation of intramolecular disulfide bonds (Figure 1A). In addition to EGF, we also isolated grass carp ErbB1a from the pituitary using a similar approach. The full-length grass carp ErbB1a cDNA is 3759 bp encoding 1253 amino acids (GenBank No.: MH161173). In silico protein modeling using SWISS-MODEL program also confirms that the 3-D structures of grass carp EGF and ErbB1a are highly comparable with that of its mammal counterpart (Figure 1B). In various brain areas, EGF was highly detected in the spinal cord, hytophalamus, medulla oblongata, cerebellum, optic tectum and olfactory bulb, with a lower extent in telencephalon (Figure 1C). For ErbB1a, it was highly detected in gill, kidney, liver, gonad, pituitary and cerebellum (Figure 1C). In addition to EGF and ErbB1a, we also cloned two HB-EGF isoforms (HB-EGFa and HB-EGFb), one BTC, ErbB1b, two ErbB2 (ErbB2a and ErbB2b), two ErbB3 (ErbB3a and ErbB3b) and two ErbB4 (ErbB4a and ErbB4b). Tissue distribution displayed that EGF, HB-EGFa, ErbB1a, ErbB1b, ErbB2a, ErbB2b and ErbB3b could all be detected in grass carp pituitary (Figure 1C).

### 2.2. Pituitary Hormone Regulation by EGF In Vitro and In Vivo

**Figure 2:**
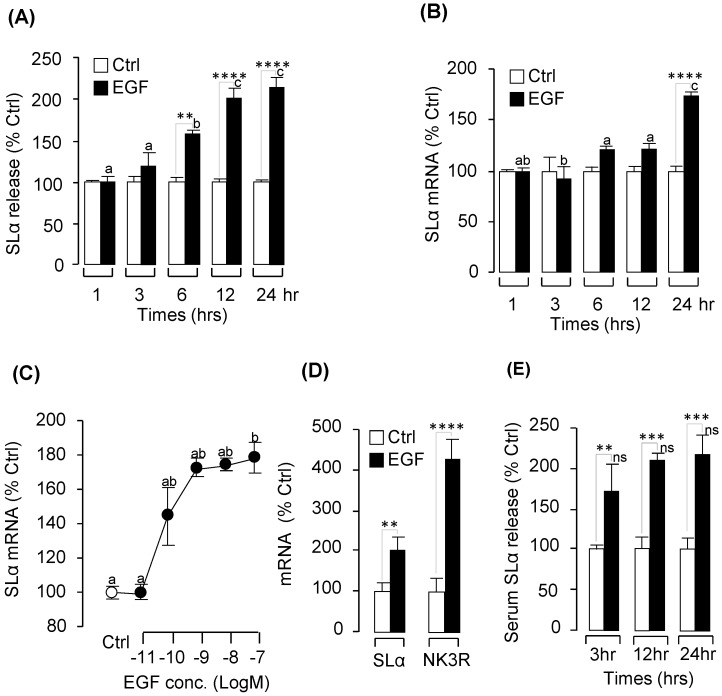
**EGF-induced grass carp** somatolactin α **(SLα) secretion and mRNA expression in vitro and in vivo.** Time course of human EGF (100 nM) treatment on SLα secretion (**A**) and mRNA expression (**B**) in grass carp pituitary cells. (**C**) Dose-dependence of 24-h treatment with increasing levels of EGF (0.01–100 nM) on SLα secretion and mRNA expression in grass carp pituitary cells (the white circle represent the control group, and the black circles are the EGF-treatment groups). In vivo effects of EGF (2 ng/g body weight) on pituitary SLα mRNA expression (**D**) and serum SLα secretion (**E**) in prepuberty grass carp. In these experiments, one-way (**C**,**D**) and two-way (**A**,**B**,**E**) ANOVA were used to test for significant differences in the control group and EGF-treatment group. ** *p* < 0.01, *** *p* < 0.001, **** *p* < 0.0001, “ns” was used to present that there were no significant differences among the EGF-induced SLα secretion at 3 h, 6 h and 24 h. The different lower-case letters were used to reveal the significant differences between the EGF-treatment group and the control group (*p* < 0.05).

As a first step to study the pituitary actions of EGF, grass carp pituitary cells were incubated for 24-h with EGF (0.1 μM). Based on the results of real-time PCR measurement, EGF treatment could elevate SLα mRNA expression without altering GH, GtHα, TSHβ, SLβ and FSHβ transcript expression (Supplemental Figure S2). Following the single dose experiment, time course experiment was performed and the results revealed that EGF treatment (100 nM) could elevate SLα secretion (Figure 2A) and mRNA expression (Figure 2B) in a time-dependent manner. To further evaluate the dose-dependence of these stimulatory effects on SLα mRNA expression, grass carp pituitary cells were exposed to increasing concentrations (0.01 nM–100 nM) of EGF separately for 24 h. In this case, EGF treatment could consistently up-regulated SLα mRNA levels in a dose-dependent fashion (Figure 2C). In the same experiment, increasing levels of EGF were both ineffective in altering transcript expression of GH, GtHα, TSHβ, SLβ and FSHβ in grass carp pituitary cell even up to 1 μM concentration.

Using prepubetal grass carp as a model, we also tested the biological function of EGF in vivo. The results demonstrated that intraperitoneal (IP) injection of EGF (2 ng/g BW) could significantly induce SLα mRNA expression in prepubertal grass carp pituitary after 24-h treatment (Figure 2D). In parallel experiments, EGF could also induce serum SLα secretion from 3 to 24 h (Figure 2E).

### 2.3. Receptor Specificity and Signal Pathway for SLα Regulation by EGF

**Figure 3:**
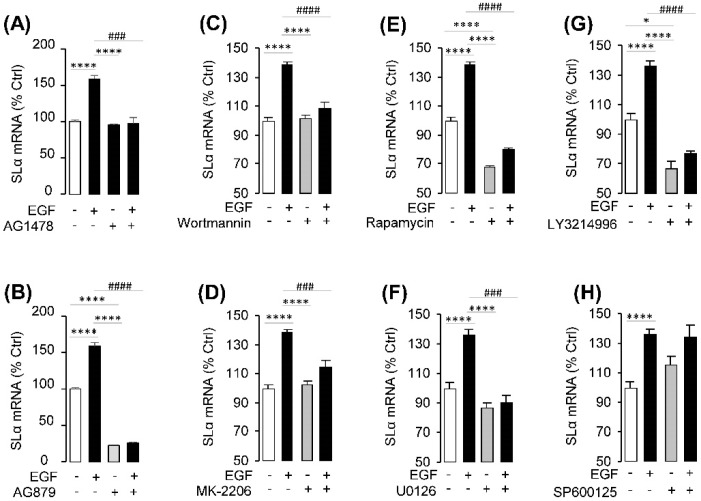
Receptor specificity and post receptor signal pathway of EGF-induced SLα mRNA expression in grass carp pituitary cells. (**A**,**B**) Effects of ErbB1 antagonist AG1478 and ErbB2 antagonist AG879 on EGF-induced SLα mRNA expression, respectively. Grass carp pituitary cells were treated for 24 h with EGF (10 nM) in the presence or absence of AG1478 (5 μM) or AG879 (5 μM). (**C**–**E**) Effects of 24-h co-treatment with the PI3K inhibitor wortmannin (1 μM), Akt inhibitor MK-2206 (10 μM) and mTOR inhibitor rapmycin (20 nM) on EGF (10 nM)-induced SLα mRNA expression, respectively. (**F**–**H**) Effects of 24-h co-treatment with the MEK inhibitor U0126 (10 μM), ERK inhibitor LY3214996 (10 μM) or JNK inhibitor SP600125 (10 μM) on EGF (10 nM)-induced SLα mRNA expression, respectively. After drug treatment, total RNA was isolated for real-time PCR of SLα. In these experiments, the two-way ANOVA was used to test the significant differences among various groups. The asterisk was used to reveal the significant difference between the EGF- or each signal pathway inhibitor-treated group, and the control group (* *p* < 0.05; ** *p* < 0.01; *** *p* < 0.001; **** *p* < 0.0001). The octothorpe was used to present the significant difference among the EGF-treated group, signal pathway inhibitor-treated group and EGF + signal pathway inhibitor-treated group (# *p* < 0.05; ## *p* < 0.01; ### *p* < 0.001; #### *p* < 0.0001).

In this experiment, a pharmacological approach was used to clarify the receptor specificity for SLα regulation by EGF. Pituitary cells were incubated for 24 h with EGF (10 nM) with simultaneous treatment of the ErbB1 antagonist AG1478 (5 μM) or ErbB2 antagonist AG879 (5 μM), respectively. Similar to the results of proceeding studies, EGF could significantly induce SLα mRNA expression. Their stimulatory effects on SLα mRNA expression could be both blocked by co-treatment with the ErbB1 antagonist AG1478 or ErbB2 antagonist AG879, respectively (Figure 3A,B). In addition, the AG879 (ErbB2 inhibitor) alone could significantly inhibit SLα mRNA expression, which indicated that ErbB2 inhibitor could also block the endogenic EGF- or HB-EGF-induced SLα expression in the pituitary (Figure 3B).

To further elucidate the signal transduction for SLα regulation by EGF, several signal inhibitors were used to co-treat with EGF in grass carp pituitary cells. As shown in Figure 3, co-treatment with the PI3K inhibitor wortmannin (1 μM) (Figure 3C), Akt inhibitor MK-2206 (10 μM) (Figure 3D) or mTOR inhibitor rapamycin (20 nM) (Figure 3E) were all effective in blocking the stimulatory effects of EGF (10 nM) on SLα mRNA expression (*p* < 0.05). Furthermore, in grass carp pituitary cells, EGF-induced SLα mRNA expression could also be suppressed by simultaneous treatment with the MEK inhibitor U0126 (10 μM) (Figure 3F) and ERK inhibitor LY32146996 (10 μM) (Figure 3G), respectively. However, the JNK inhibitor SP600125 (10 μM) could not block EGF-induced SLα mRNA expression in grass carp pituitary cells (Figure 3H).

### 2.4. EGF-Induced NK3R Expression in Grass Carp Pituitary Cells

**Figure 4:**
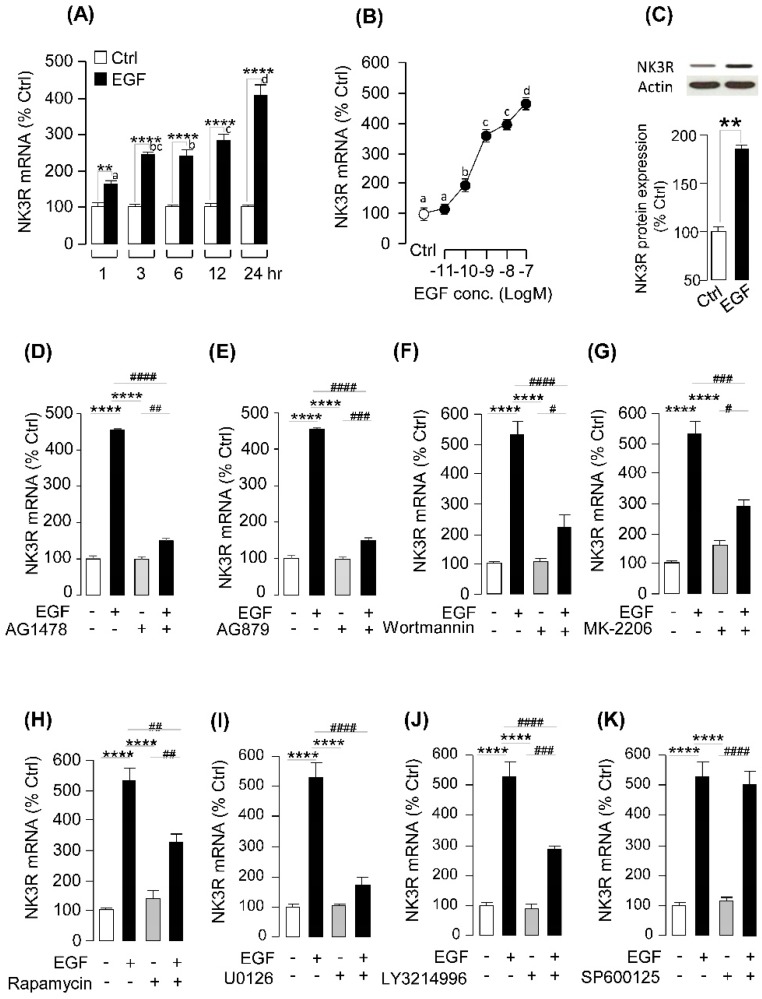
**EGF-induced NK3R expression in grass carp pituitary cells.** (**A**) Time course of EGF (100 nM) treatment on NK3R expression in grass carp pituitary. (**B**) Dose-dependence of 24-h treatment with increasing levels of EGF (0.01–100 nM) on NK3R mRNA expression (the white circle is the control group, and the black circles are the EGF-treatment group). (**C**) Effect of EGF (100 nM) on the protein production of NK3R in grass carp pituitary cells. (**D**,**E**) Effects of ErbB1 antagonist AG1478 and ErbB2 antagonist AG879 on EGF-induced NK3R mRNA expression, respectively. Grass carp pituitary cells were treated for 24 h with EGF (10 nM) in the presence or absence of AG1478 (5 μM) or AG879 (5 μM), respectively. (**F**–**H**) Effects of 24-h co-treatment with the PI3K inhibitor wortmannin (1 μM), Akt inhibitor MK-2206 (10 μM) and mTOR inhibitor rapmycin (20 nM) on EGF (10 nM)-induced NK3R mRNA expression, respectively. (**I**–**K**) Effects of 24-h co-treatment with the MEK inhibitor U0126 (10 μM), ERK inhibitor LY3214996 (10 μM) or JNK inhibitor SP600125 (10 μM) on EGF (10 nM)-induced NK3R mRNA expression, respectively. In these experiments, one-way (**A**–**C**) and two-way (**D**–**K**) ANOVA were used to test for significant differences among various groups. (* *p* < 0.05; ** *p* < 0.01; ** *p* < 0.001; **** *p* < 0.0001). The asterisk or lower-case letters were used to reveal the significant difference between EGF- or each signal pathway inhibitor-treated group, and the control group (* *p* < 0.05; ** *p* < 0.01; *** *p* < 0.001; **** *p* < 0.0001). The octothorpe was used to present the significant difference among the EGF-treated group, signal pathway inhibitor-treated group and EGF + signal pathway inhibitor-treated group (# *p* < 0.05; ## *p* < 0.01; ### *p* < 0.001; #### *p* < 0.0001).

To examine the direct effect of EGF on NK3R expression at the pituitary level, primary cultured grass carp pituitary cells were challenged with human EGF. The time-course experiment revealed that EGF (100 nM) could significantly stimulate NK3R mRNA expression from 1 h to 24 h in a time-dependent manner (Figure 4A). In the parallel dose-dependent studies, a 24 h incubation with increasing levels of EGF (0.01–100 nM) could also trigger NK3R mRNA expression in a concentration-related fashion (Figure 4B). At the protein level, WB result demonstrated that EGF (10 nM) could significantly increase NK3R cell content in grass carp pituitary cells (Figure 4C). To further clarify the receptor specificity for NK3R regulation by EGF, a pharmacological approach was used. As shown in Figure 4D,E, the stimulatory effects of EGF on NK3R mRNA expression was abolished by simultaneous treatment with the ErbB1 inhibitor AG1478 or ErbB2 inhibitor AG879, respectively. With the using of pharmacological blockers targeting different signaling pathways, the signal transduction mechanisms for NK3R expression regulated by EGFR activation was examined. As shown in Figure 4F–H, the stimulatory effects on NK3R mRNA expression induced by EGF treatment were notably suppressed by simultaneous incubation with the PI3K inhibitor wortmannin (1 μM), Akt inhibitor MK-2206 (10 μM) or mTOR inhibitor rapmycin (20 nM) (*p* < 0.05). Furthermore, co-treatment with the MEK inhibitor U0126 (10 μM), or ERK inhibitor LY3214996 (10 μM) could also inhibit EGF-induced NK3R mRNA expression in carp pituitary cells (Figure 4I,J). However, JNK inhibitor SP600125 (10 μM) could not block EGF-induced NK3R mRNA expression in grass carp pituitary cells (Figure 4K).

### 2.5. Synergistic Effects of EGF and NKB on SLα mRNA Expression

**Figure 5:**
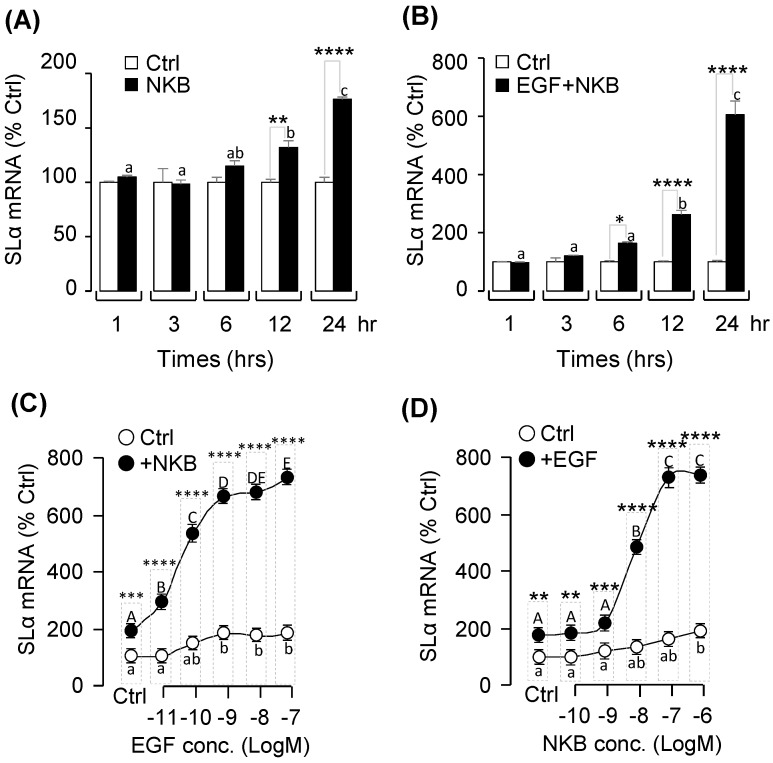
**Synergistic effects of neurokinin B (NKB) with EGF on SLα mRNA expression.** (**A**,**B**) Time course of NKB (1 μM), and NKB (1 μM) + EGF (10 nM) treatment on SLα mRNA expression. (**C**) Effect of EGF concentration (0.01–100 nM) on basal and NKB (1 μM)-induced SLα mRNA expression in grass carp pituitary cells. (**D**) Effect of NKB concentration (0.1–1000 nM) on basal and EGF (10 nM)-induced SLα mRNA expression in grass carp pituitary cells. After drug treatment, total RNA was isolated for real-time PCR of SLα mRNA expression. In these experiments, the two-way ANOVA was used to test for significant differences among various groups. * *p* < 0.05, ** *p* < 0.01, *** *p* < 0.001, **** *p* < 0.0001. The different capital letters were used to reveal the significant difference between EGF + NKB (1000 nM)-treated group and the control group (*p* < 0.05). The different lower-case letters were used to present the significant difference between EGF-treated group and the control group (*p* < 0.05).

To further examine the functional role of EGF-induced NK3R gene expression on SLα expression, co-treatment of EGF with NKB was performed in grass carp pituitary cells. As shown in Figure 2B and Figure 5A, static incubation with EGF and NKB alone were both effective in increasing SLα mRNA levels in carp pituitary cells in a time-dependent manner. Interestingly, the stimulatory effect on SLα mRNA expression was markedly enhanced (up to 6 folds basal) especially after 12–24 h of drug treatment with simultaneous exposure to both EGF and NKB (Figure 5B). In parallel studies, the dose-dependence of this synergistic action was also confirmed. In this case, NKB-induced SLα mRNA expression was found to be aggravated in a concentration-related fashion with simultaneous treatment with increasing levels of EGF (0.01–100 nM; Figure 5C). Similar dose-dependence of the potentiating effect was also noted in the reciprocal experiment with co-treatment of EGF with increasing concentrations of NKB (0.1–1000 nM; Figure 5D).

**Figure 6:**
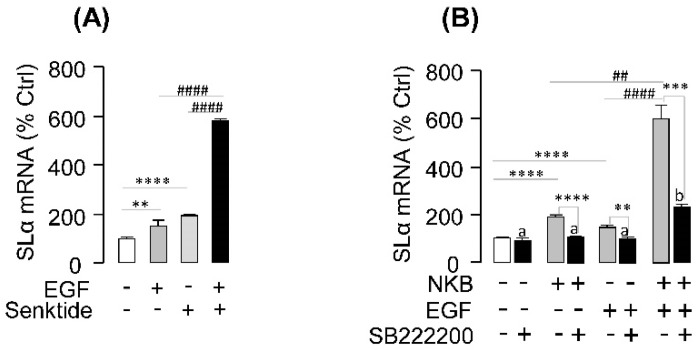
**Synergistic effects of EGF with NK3R agonist and antagonist.** (**A**) EGF (10 nM) synergizes with NK3R agonist senktide (1 μM) to stimulate SLα mRNA expression in grass carp pituitary cells. (**B**) Effects of NK3R antagonist SB222200 on EGF, NKB or EGF + NKB-induced SLα mRNA expression. In this experiment, grass carp pituitary cells were treated for 24-h with NKB (1 μM), EGF (10 nM) and EGF (10 nM) + NKB (1 μM) in the presence or absence of NK3R antagonist SB222200 (10 μM). After drug treatment, total RNA was isolated for real-time PCR of SLα mRNA expression. In these experiments, the two-way ANOVA was used to test for significant differences among various groups. The asterisk was used to show the significant difference between the EGF or senktide treatment group and the control group (* *p* < 0.05; ** *p* < 0.01; *** *p* < 0.001; **** *p* < 0.0001), the octothorpe or different lower-case letters were recruited to present the significant difference between the EGF+senktide treatment group and EGF/senktide alone treatment group (# *p* < 0.05; ## *p* < 0.01; ### *p* < 0.001; #### *p* < 0.0001).

To establish the functional link between EGF potentiation and NK3R expression at the pituitary level, the NK3R agonist senktide was substituted for NKB in the potentiation study with EGF co-treatment. In this case, NK3R activation with senktide was found to mimic the synergistic effects of NKB on SLα mRNA expression when given together with EGF (Figure 6A). Furthermore, co-treatment with NK3R antagonist SB222200 could block the synergistic effects of EGF and NKB on SLα mRNA expression in grass carp pituitary cells (Figure 6B). These results indicate that the synergistic effect of EGF and NKB on SLα mRNA expression is dependent on NK3R activation.

### 2.6. Signal Transduction for the Synergistic Effects of EGF and NK3R Activation

**Figure 7:**
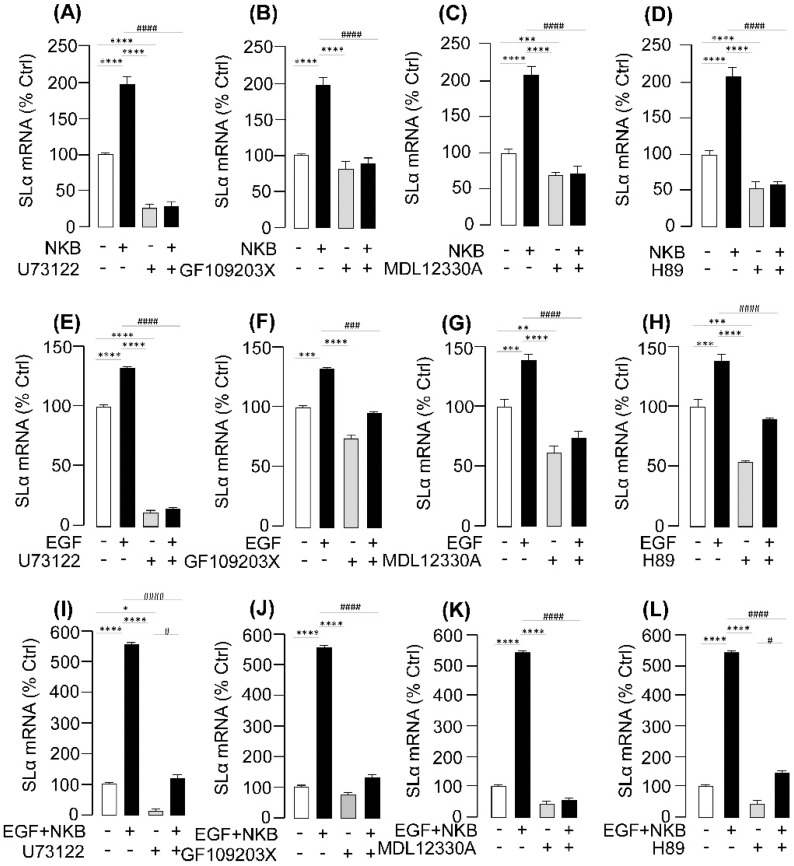
**Signal transduction mechanisms of EGF potentiation of NKB-induced SLα mRNA expression.** (**A**) Effects of 24-h treatment with the PLC inhibitor U73122 (10 μM) or PKC inhibitor GF109203X (10 μM) on EGF (10 nM), and NKB (1 μM) + EGF (10 nM)-induced SLα mRNA expression were examined in grass carp pituitary cells. (**B**) Effects of 24-h co-treatment with AC inhibitor MDL12330A (10 μM) or PKA inhibitor H89 (10 μM) on NKB (1 μM) + EGF (10 nM)-induced SLα mRNA expression in grass carp pituitary cells. After drug treatment, total RNA was isolated for real-time PCR of SLα mRNA expression. In these experiments, the two-way ANOVA was used to test for significant differences among various groups. The asterisk was used to show the difference between single-treating groups and the control group (* *p* < 0.05; ** *p* < 0.01; *** *p* < 0.001; **** *p* < 0.0001). The octothorpe was recruited to present the significant difference between the EGF- or EGF+NKB-treated group and EGF+ or EGF+ NKB+ signal pathway inhibitors combined groups (# *p* < 0.05; ## *p* < 0.01; ### *p* < 0.001; #### *p* < 0.0001).

To further evaluate if EGF co-treatment can potentiate the stimulatory effects on SLα gene expression mediated through the cAMP-, and PKC-dependent cascades, pituitary cells were also challenged with EGF in the presence of various stimulators for the respective signaling cascades. In these cases, pituitary cells were exposed to EGF and NKB alone or in combination with/without co-treatment with various inhibitors targeting the AC/cAMP/PKA, and PLC/IP_3_/PKC pathways. The results demonstrated that simultaneous incubation with the PLC inhibitor U73122 (Figure 7A,E), PKC inhibitor GF109203X (Figure 7B,F), AC inhibitor MDL12330A (Figure 7C,G), PKA inactivator H89 (Figure 7D,H), not only could attenuated the stimulatory action on SLα mRNA expression induced by NKB or EGF treatment alone (*p* < 0.05), but also notably suppressed the synergistic effect of SLα mRNA expression caused by EGF and NKB co-treatment in grass carp pituitary cells (Figure 7I–L).

## 3. Discussion

In the present study, to shed light on the pituitary actions of EGF in fish models, grass carp EGF and ErbBs were cloned and phylogenetic analysis based on their cDNA sequence has confirmed that they are the orthologue of vertebrate EGF and EGFR, and closely related to EGF and EGFR identified in zebrafsih, respectively [33]. In grass carp, EGF was also found to be expressed in the pituitary. In addition, we also isolated two isoforms of HB-EGF, namely HB-EGFa and HB-EGFb, which are also highly detected in grass carp pituitary. Of note, similar to zebrafish [17], ErbB1a, ErbB1b, ErbB2a, ErbB2b, ErbB3a, ErbB3b, ErbB4a, and ErbB4b could also be detected in grass carp pituitary. Together with the high detection of EGF, HB-EGF and their receptors in grass carp pituitary, these findings raise the possibility that EGF may act in an autocrine/paracrine manner to regulate pituitary functions in grass carp.

In mammals, ErbBs and their ligands are expressed in pituitary lacto-somatotroph cells and also have a functional role in prolactin regulation at both gene and protein levels, leading to changes in prolactin transcription and synthesis [34,35], pituitary proliferation, and hormonal secretion [13,14,15]; thus far, EGF has been shown to have little effect on growth hormone (GH) regulation [16,36]. In grass carp pituitary cells, we found that EGF could significantly induce SLα secretion and mRNA expression in a time- and dose-dependent manner. Furthermore, IP injection of EGF could also induce serum SLα secretion and pituitary SLα mRNA expression in grass carp. Similar to prolactin, SLα is also a member of GH/PRL family with several functions in fish models, including background adaption, reproduction, acid-base balance, lipid metabolism and immune responses [37]. Two isoforms of somatolactin, SLα and SLβ, have been identified in fish pituitary, e.g., in zebrafish [19] and grass carp [21], and suspected to have overlapping and yet distinct functions. In the present study, EGF could only induce pituitary SLα secretion and mRNA expression, but no effect on SLβ secretion and mRNA expression in vivo and in vitro.

The biological effect of EGF on target tissues is mediated by homodimerization of ErbB1 or via heterodimerization with other ErbB family members, with ErbB2 being the favored dimerization partner [38,39,40,41]. ErbB2 enhances the ligand affinity, as well as ligand-induced phosphorylation of ErbB receptors, prevents receptor internalization, and prolongs signal duration [42,43,44,45,46]. In our present study, ErbB1a/b and ErbB2a/b were all abundantly detected in grass carp pituitary. The stimulatory actions on SLα mRNA expression induced by EGF could both be blocked by ErbB1 and ErbB2 antagonists, respectively. These results, as a whole, suggested that EGF can act at the pituitary level to induce SLα secretion and synthesis by ErbB1 and ErbB2 heterodimerization.

In mammals, NK3R can preferentially bind with NKB, but to a lower extent for other tachykinins, and its activation is responsible for a wide range of biological actions, including reproductive regulation, gut motility and secretory functions [47]. In our previous studies, grass carp NKB was found to stimulate SLα secretion and gene expression in carp pituitary cells via NK3R activation and subsequent stimulation of cAMP/PKA, PLC/IP_3_/PKC and Ca^2+^/CaM/CaMK-II pathways [25]. Although NK3R is known to be important in reproductive functions in mammals, not much is known regarding the neuroendocrine regulation of NK3R expression at the pituitary level. Except for a single report in zebrafish suggesting that brain NK3R gene expression can be modified by estrogen treatment [29], to our knowledge, no information is available for NK3R regulation in the fish model, especially at the pituitary level as a regulatory target for modulation of pituitary hormone secretion/gene expression. In the present study, we first reported that EGF could induce pituitary NK3R expression in grass carp pituitary cells via ErbB1 and ErbB2 activation and subsequent stimulation of PI3K/Akt/mTOR, and MEK/ERK pathways.

Given our observations that (i) NK3R and ErbB activations are both reported to regulate SLα expression, and (ii) EGF could significantly induce NK3R expression in grass carp pituitary cells, it is reasonable for us to speculate that EGF-induced NK3R expression might be a mechanism for amplifying the NKB-induced SLα responses. This hypothesis is also consistent with the observations that (i) NK3R gene expression induced by ErbB activation (first observed at 1 h after EGF treatment) occurred prior to the onset of the potentiating effect on SLα mRNA expression caused by EGF and NKB co-treatment (first observed only after 6 h with drug treatment), (ii) co-treated with senktide, a NK3R agonist, was effective in inducing the potentiating action similar to NKB when given together with EGF, (iii) the synergistic effect on SLα gene expression induced by NKB and EGF co-treatment could be partially suppressed by the NK3R antagonist SB222200, and (iv) similar potentiating effect caused by senktide+EGF was also sensitive to pharmacological blockade of the post-receptor signaling cascades, including AC/cAMP/PKA blockade using the AC inhibitor MD12330A, PKA inhibitor H89, PLC/IP_3_/PKC blockade using the PLC inhibitor U73122, and PKC inactivator GF109203X. These findings, taken together, provide evidence that the synergism between EGF and NKB on SLα gene expression is dependent on NK3R expression at the pituitary level and subsequent activation of post-receptor signaling cascades functionally coupled with NK3R activation.

**Figure 8:**
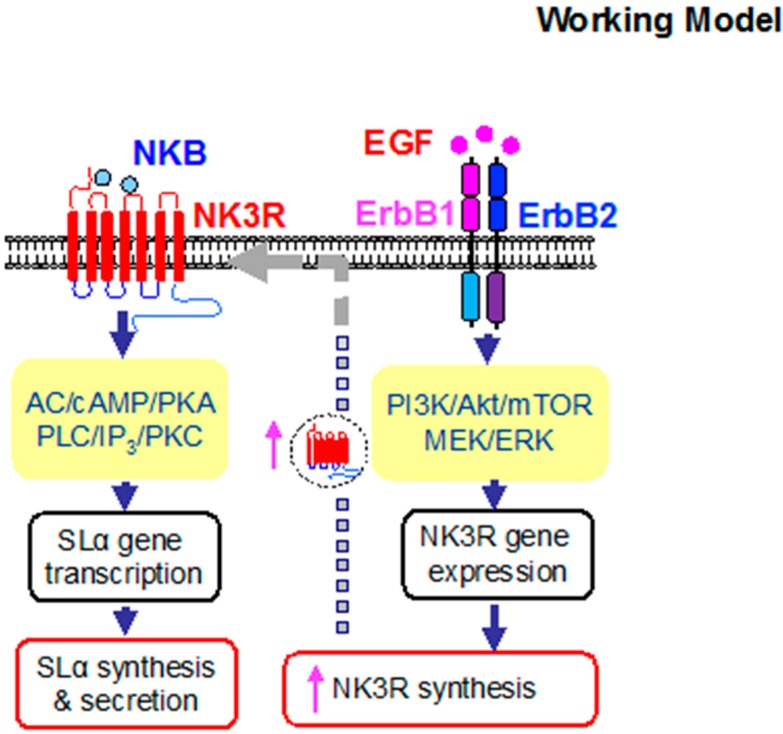
**Working model of the interaction between EGF and NKB on SLα mRNA expression in carp pituitary cells.** In grass carp pituitary cells, EGF could elevate via activation of ErbB1 and ErbB2 by functional coupling with PI3K/Akt/mTOR, MEK/ERK pathways. The stimulation of NK3R gene expression could contribute to the potentiating effect on NKB-induced SLα mRNA expression, which were mediated by NK3R via functional coupling with the AC/cAMP/PKA and PLC/IP_3_/PKC signaling pathways.

In summary, we have cloned the grass carp EGF and ErbB, and confirmed their expression in grass carp pituitary. At the pituitary level, EGF could significantly induce pituitary SLα and NK3R secretion and mRNA expression via ErbB1 and ErbB2 activation and subsequent stimulation of PI3K/Akt/mTOR, and MEK/ERK pathways. In our recent study, we have reported that NKB could activate NK3R to induce SLα secretion and mRNA expression in grass carp pituitary cells. Interestingly, in the present study, we further found that EGF could significantly enhance NKB-induced SLα mRNA expression. The synergistic effect of EGF and NKB on SLα gene expression is highly dependent on NK3R expression at the pituitary level and the post-receptor signaling cascades coupled to NK3R, including the AC/cAMP/PKA and PLC/IP_3_/PKC pathways (Figure 8). Our findings for the first time demonstrate that functional interactions between EGF and NKB occur in the fish pituitary and play a role in SLα gene expression.

## 4. Materials and Methods

### 4.1. Animals and Reagents

Healthy grass carps (*Ctenopharyngodon idellus*, ~18 months-old) with body weight ranging from 1.5–2.0 kg were acquired from local markets and maintained in 250-L aquaria under 12D:12L photoperiod at 20 ± 2 °C for two weeks. Since the grass carp at this stage was prepubertal and sexual dimorphism was not apparent, mixed sexual carps were used for primary pituitary cell culture according to the protocol approved by the committee for animal use at Huazhong Agricultural University (Ethical Approval No. HBAC20091138; Date: 15 November 2009). Grass carp NKB (EMHDIFVGLM-NH_2_) synthesized by GenScript Corporation (Piscataway, NJ) was dissolved in DMSO and stored frozen at −80 °C as 1 mM stocks in small aliquots. Human EGF was purchased from GenScript Corporation (Piscataway, NJ, USA) and dissolved in double-distilled deionized water and stored as 0.1 mM stocks in small aliquots at −80 °C. Other drugs for receptor specificity and post-receptor signal pathway are listed in Supplemental Table S1. In our Western blot and FIA studies, the antibodies used for the detection of grass carp NK3R and SLα were synthesized by our lab (Supplemental Table S2). The second antibodies (raised in mouse or rabbit) conjugated with horseradish peroxidase were purchased from KPL (Gaitherbug, MD, USA).

### 4.2. Molecular Cloning, Tissue Expression and Structural Analysis of carp EGF and EGFR

Total RNA prepared from grass carp pituitary was reversely transcribed using Hifair^TM^ III 1st Strand cDNA Synthesis Kit (gDNA digester plus) (Yeasen Biotech Co. Ltd., Shanghai, China). Based on the grass carp transcriptomic sequence, primers were designed to amplify the conserved region of EGF and ErbBs transcripts in grass carp. Full-length cDNA for grass carp EGF was then compiled using the MacVector 9.5.2 software (Oxford Molecular, Madison, WI, USA). Phylogenetic analysis based on EGF and ErbB nucleotide sequences was conducted with MEGA 6.0 using the neighbor-joining method [48]. The 3-D protein models of EGF and EGFR were constructed SWISS-MODEL server [49]. For tissue and brain distribution of EGF and EGFR in grass carp, transcriptomic analysis and RT-PCR was conducted in RNA isolated from various tissues and selected brain areas (n = 3) using primers specific for carp EGF, HB-EGF, BTC, and ErbBs (Supplemental Table S3). In these studies, RT-PCR of β-actin was performed to serve as an internal control.

### 4.3. Measurement of Pituitary Hormone mRNA Expression

Following the previous studies [50], primary cultured grass carp pituitary cells were dispersed by trypsin/DNase II digestion method. The cells were counted by microscopy and hemocytometer. Then the cells were seeded in 24-well plates (with poly-D-lysine coating) at ~2.5 × 10^6^ cells/ml/well. After 24 h, the old plating medium was replaced by the testing medium with several drugs, and the cells were then incubated at 28 °C for the duration as indicated. After drug treatment, total RNA was isolated from the individual well using TRIeasy^TM^ Total RNA Extraction Reagent and reversely transcribed by Hifair^TM^ III 1st Strand cDNA Synthesis Kit (gDNA digester plus) (Yeasen Biotech Co. Ltd., Shanghai, China). The RT samples obtained were subjected to qPCR using a LightCycler SYBR Green I Kit (Roche, Stockholm, Sweden) with primers specific for grass carp SLα and NK3R (Supplemental Table S3), respectively. In these studies, serial dilutions of plasmid DNA containing the ORF of SLα (GeneBank no.: EF372074) and NK3R (GenBank no: JQ254913) cDNA were used as the standards for data calibration. Parallel real-time PCR measurement of β-actin was also conducted in the individual experiment to serve as the internal control.

### 4.4. Measurement of SLα Secretion by Fluorescence-Based ELISA

The primary cultured pituitary cells from prepubertal grass carp were incubated with several drugs, respectively. Then the culture medium was collected for measurement of SLα release using fluorescence-based ELISA. In this study, the recombinant grass carp SLα protein was synthesized and used to product the polyclonal antibody. Then the recombinant SLα protein was biotinylated and used as the tracer for the respective assays. Protein A (0.5 μg/mL) precoated Costar 96-well black plate (Thermo Fisher, MA) was used to load the protein samples, biotinylated SLα (12.5 ng/mL), and SLα antibody (0.45 μg/mL) (For information of SLα antibody, please refer to Supplemental Table S2). After overnight incubation at 4 °C, washing buffer was used to rinse each well to remove non-specific binding of the primary antibody. Then we introduced HRP-conjugate streptavidin (0.5 μg/mL) to incubate each well for anther 1 h at room temperature. After that, unbound second antibody was removed by decanting and washing three times. Then a 100 μL volume of QuantaBlu^TM^ Fluorogenic Peroxidase Substrate (Thermo Scientific, Rockford, USA) was then added into each well for signal development. Finally, FluoStar OPTIMA-Fluorecence plate reader (BMG Labtech GmbH, Ortenberg, Germany) was used to detect the fluorescence signals.

### 4.5. In Vivo EGF Treatments and Sampling Procedure

After entraining the fish in 250-L tanks with the one-meal-per-day feeding schedule, drug treatment by intraperitoneal (IP) injection was performed as described previously [51]. Twenty-four prepubertal grass carps (body weight (BW): 850 ± 75 g) were divided into two experimental groups (n = 12 carps/group). Each grass carp received one intraperitoneally injection of 2 ng EGF/g BW suspended in 0.15 M NaCl or vehicle alone (control). After treatment, the blood from each fish were collected in 3 h, 6 h and 24 h, respectively, by using the vacuum blood collection tube. After 24 h, the grass carps were sacrificed by anesthesia, and the pituitary was harvested from each fish and stored in liquid nitrogen until the mRNA extraction.

### 4.6. Western Blot for Signaling Kinases

To detect NK3R protein synthesis, the primary cultured pituitary cells derived from grass carp were treated with EGF (100 nM) for 24 h. Then the culture medium from each well was removed, and remaining cells were rinsed with PBS and lysed in RIPA buffer (50 mM Tris.HCl, 150 mM NaCl, 1 mM EDTA, 1% NP-40, and 0.25% Na deoxycholate) containing a final concentration of 1× protease inhibitor cocktail (Roche). The cells lysate was cleared by high-speed centrifugation at 4 °C, and the clear supernatant were resolved in 10% gel by SDS-PAGE, and Western blotting was conducted according to the procedures in our previous described [51]. The working concentration of grass carp NK3R antibody was 0.584 μg/mL. Following the incubation, the membranes were washed three times to remove non-specific binding of primary antibodies and the HRP-conjugated secondary antibodies [goat anti-rabbit IgG (1:5000)] were introduced for signal development. Chemiluminescence signals for target immune-reactivity were detected using SuperSignal West Pico (PIERCE, Rockford, IL, USA) as the substrate and quantified using the IC440 CF Digital Science Image Station (Kodak, Rochester, NY, USA). In these experiments, western blot of β-actin was used as an internal control using its antibody (1:15,000; Oncogen Co., Cambridge, MA, USA).

### 4.7. Data Transformation and Statistics

For SLα fluorescence immunoassays, standard curves with a range from 0.98 ng/mL to 500 ng/mL and ED_50_ value of 60–80 ng/mL for SLα were used for data calibration with a four-parameter logistic equation of the GraphPad Prism 7 program (GraphPad, San Diego, CA, USA). For real-time PCR of SLα and NK3R mRNA measurement, standard curves with a dynamic range of ≥10^5^ and correlation coefficient ≥0.95 were used for data calibration with the ABI7500 software (Thermo fisher, Singapore) under unsupervised mode. Given no significant changes were noted for β-actin mRNA levels between different experiment groups in our studies, SLα and NK3R mRNA data were simply transformed as a percentage of the mean value in the control group without drug treatment (as “%Ctrl”). The data obtained from qPCR were analyzed for statistical significance using the GraphPad Prism 7 program (Graphpad, San Diego, CA, USA). The data presented (expressed as Mean ± SEM) were pooled results from 6–8 experiments Prior to statistical analysis, all data were tested for normality of distribution using the Shapiro-Wilk normality test. One-way ANOVA and two-way ANOVA were used to test the significant difference according to different experiments.

## Supplementary Materials

Supplementary materials can be found at http://www.mdpi.com/1422-0067/20/1/91/s1.

## Abbreviations

AC	Adenylyl cyclase
Akt	protein kinase B
ERK	extracellular signal-regulated kinase
EGF	epidermal growth factor
NK3R	Type 3 NK receptor
NKB	neurokinin B
PI3K	phosphatidylinositol-3-kinase
PKC	Protein kinase C
PKA	Protein kinase A
PLC	phospholipase C
SLα	Somatolactin α
