# Supplementary material for: NK3R Mediates the EGF-Induced SLα Secretion and mRNA Expression in Grass Carp Pituitary
Source: Int J Mol Sci. 2018 Dec 26;20(1):91. doi: 10.3390/ijms20010091 (PMC6337684; doi:10.3390/ijms20010091)
Supplement: Supplementary file 1 [file ijms-20-00091-s001.pdf]

Supplemental Figure 1

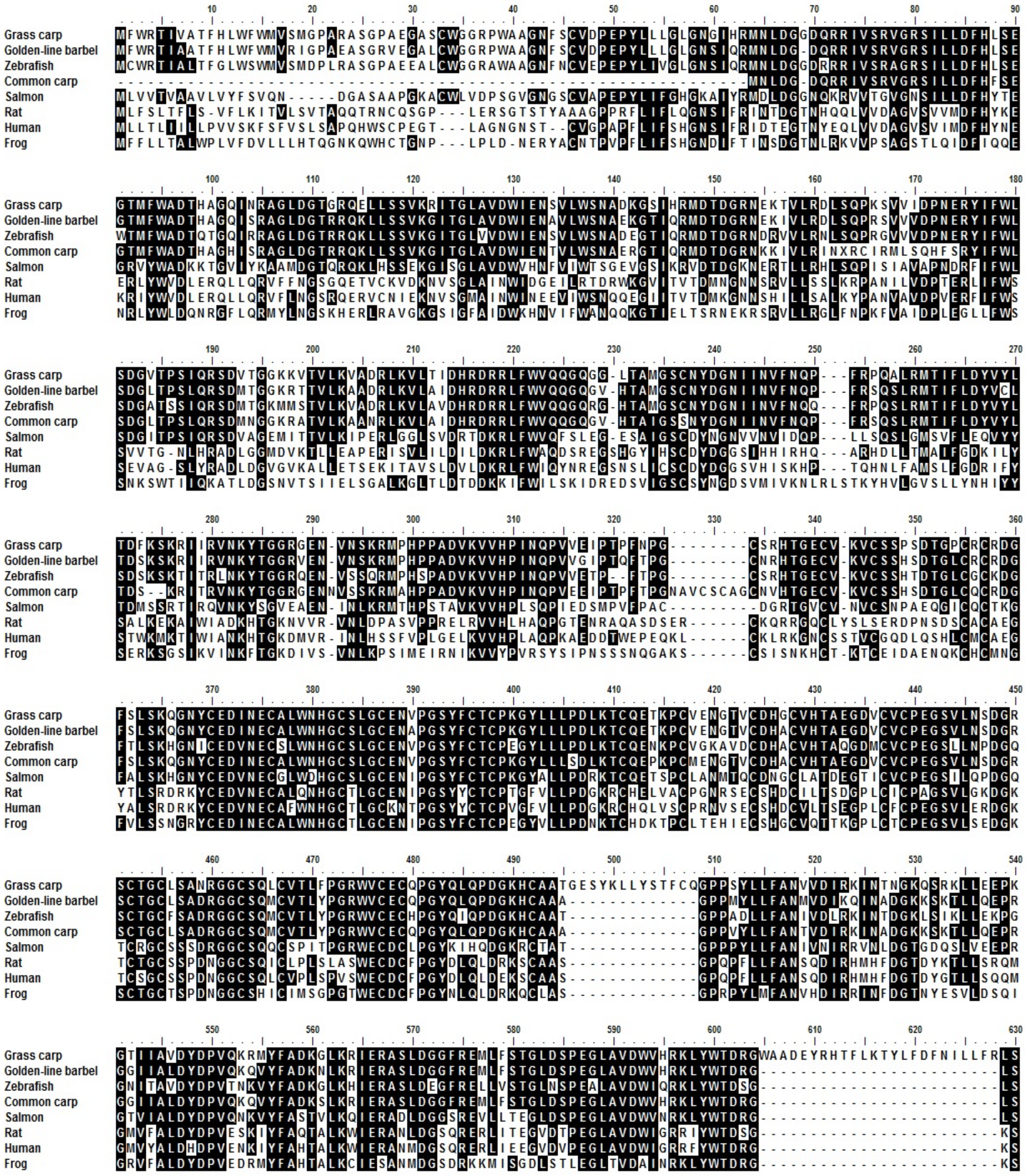

Supplemental Figure 1

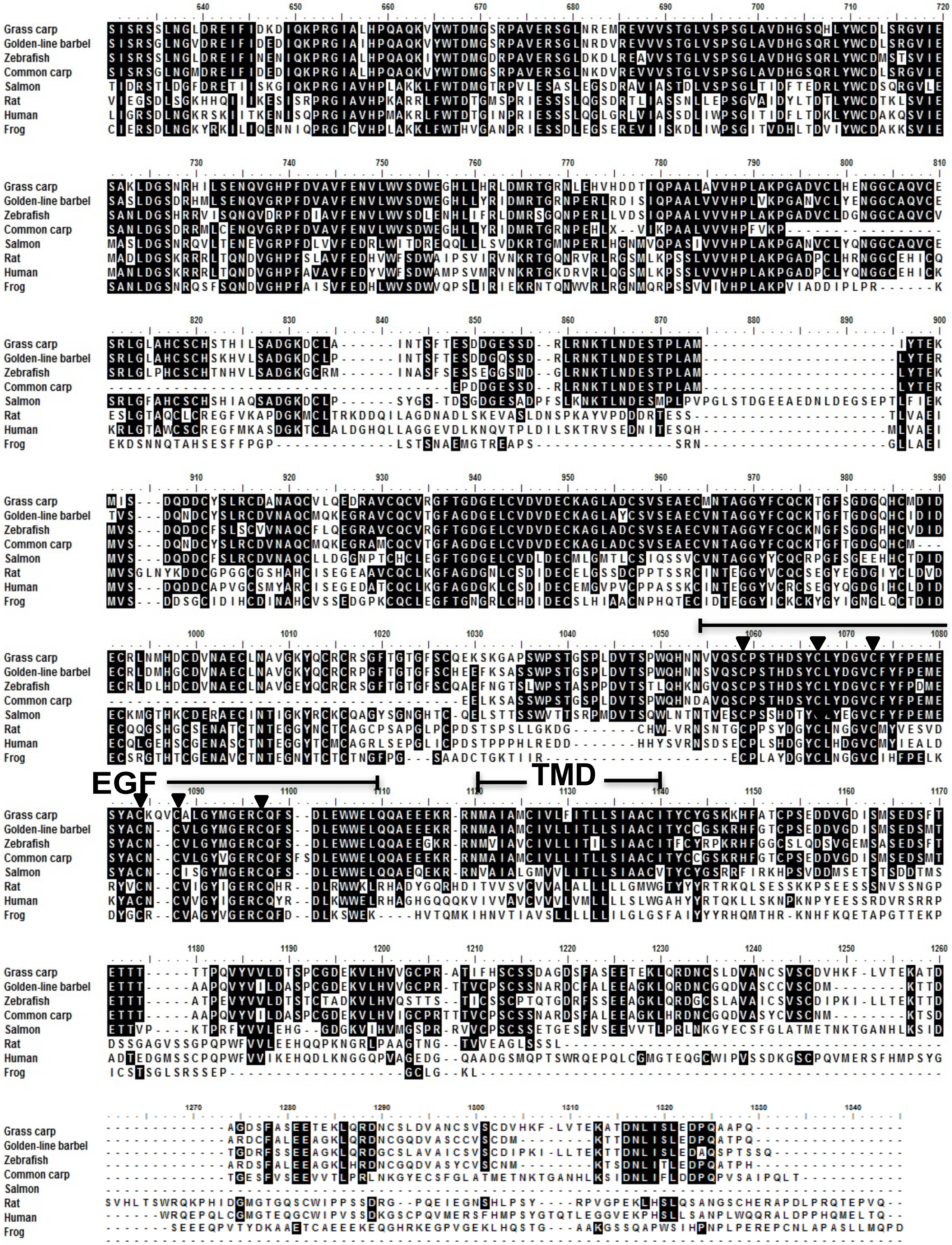

Supplemental Figure 1. Sequence alignment of EGF among grass carp and other vertebrates.

Supplemental Figure 2

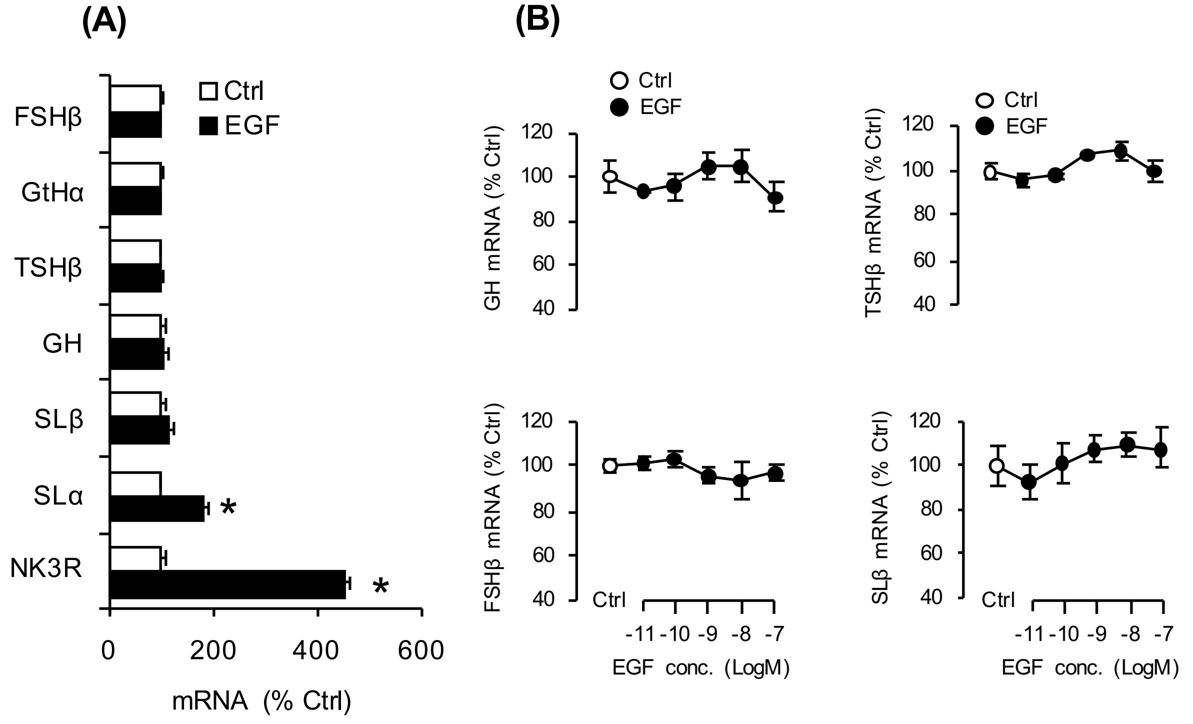

Supplemental Figure 2. Effects of Epidermal growth factor (EGF) on grass carp pituitary hormone gene expression in carp pituitary cells. (A) Effects of 24-hr treatment with carp EGF (100 nM) on pituitary hormone gene expression. (B) Dose-dependence of 24-hr treatment of carp EGF (0.01-100 nM) on pituitary hormone mRNA expression. In these studies, total RNA was extracted from pituitary cells by Trizol and reversely transcribed for real-time RT-PCR measurement of pituitary hormone mRNA.

Supplemental Table 1

The information for the drugs used in receptor specificity and signal transduction.

Item	Function	Manufacture	Cat. No.
Senktide	NK3R agonist	Tocris Bioscience	1535
SB222200	NK3R antagonist	MCE	HY-15722
Forskolin	AC activator	MCE	HY-15371
TPA	PKC activator	Tocris Bioscience	1201
MDL12330A	AC inhibitor	Merck	444200
H89	PKA inhibitor	MCE	HY-15979
GF109203X	PKC inhibitor	Sigma	B6292
U73122	PLC inactivator	Sigma	U6756
U0126	MEK1/2 inhibitor	Selleck	S1102
LY3214996	ERK1/2 inhibitor	Selleck	S8534
SP600125	JNK inhibitor	Selleck	S1460
Wortmannin	PI_3K inhibitor	MCE	HY-10197
Rapamycin	mTOR inhibitor	MCE	HY-10219
MK-2206	AKT inhibitor	Selleck	S1078
AG1478	ErbB1 inhibitor	MCE	HY-13524
AG879	ErbB2 inhibitor	MCE	HY-20878

Supplemental Table 2 Antibodies Used in Western Blot and FIA

Peptide/protein target	Antigen sequence (if known)	Name of Antibody	Manufacturer, catalog #, and/or name of individual providing the antibody	Species raised in; monoclonal or polyclonal
**Grass carp NK3R**	a synthetic peptide (KLH coupled "CESDTNANPARRKSS") in the C-terminal of grass carp NK3R.	Anti-NK3R	Prof GF Hu, HZAU, Wuhan, China	polyclonal in Rabbit
**Grass carp somatolactin alpha**	Recombinant grass carp SLα protein (GenBank No EF372074.1)	Anti-SLα	Prof GF Hu, HZAU, Wuhan, China	polyclonal in Rabbit
**β Actin**	a synthetic peptide (KLH coupled) covering the conserved region of human, rat and mouse Actin.	Anti-Actin mAB	Calbiochem, catalog #CP01	monoclonal IgM in Mouse

Abbreviations: KLH, keyhole limpet hemocyanin; mAb, monoclonal antibody.

Supplemental Table 3

Primers used for tissue distribution and quantitative real-time PCR

Target	Forward primer	Reverse primer	Length	Annealing Tm	Accession No.
**Primers for tissue distribution**					
EGF	TCTGTATGATGGAGTGTGCTT	CTGAAGAGCAAGAGTGGAA	221 bp	56℃	MH161172
ErbB1a	AGTCGCATTAGGTCTTACG	TTGAGCAGGTGTTGTGAG	222 bp	54℃	MH161173
β-Actin	CTGGTATCGTGATGGACTCT	AGCTCATAGCTCTTCTCCAG	280 bp	56℃	DQ211096
**qPCR primers**					
SLα	ACCCACTGTACTTCAATCTCC	CGTCGTAACGATCAAGAGTAG	283 bp	52℃	EF372074.1
NK3R	GCCAAGAGAAAGGTTGTGAAGA	GTGTACATGCTGCTCTGGCG	330 bp	56℃	JQ254913
β-Actin	CTGGTATCGTGATGGACTCT	AGCTCATAGCTCTTCTCCAG	280 bp	56℃	DQ211096
